# Visual and microscopic lesions of enteritis in slaughtered swine: pathogen identification and antimicrobial resistance implications

**DOI:** 10.1007/s11259-026-11200-9

**Published:** 2026-04-27

**Authors:** Luisa Zasso Neis, Rogério Oliveira Rodrigues, Angélica Cavalheiro Bertagnolli, Karine Ludwig Takeuti, Fabiana Quoos Mayer

**Affiliations:** 1 Programa de Pós-Graduação em Saúde Animal, Instituto de Pesquisas Veterinárias Desidério Finamor, Eldorado Do Sul, Rio Grande do Sul 92990-000 Brazil; 2 Centro de Pesquisa em Saúde Animal, Departamento de Diagnóstico e Pesquisa Agropecuária, Secretaria da Agricultura, Pecuária, Desenvolvimento Sustentável e Irrigação, Rio Grande Do Sul 92990-000 Eldorado Do Sul, Brazil; 3 Universidade Feevale, Novo Hamburgo, Rio Grande Do Sul Brazil; 4 Programa de Pós-Graduação em Biologia Celular e Molecular, Centro de Biotecnologia, Universidade Federal do Rio Grande do Sul, Rio Grande do Sul 91501-970 Porto Alegre, Brazil

**Keywords:** Pork carcasses, Lesion classification, One health, *E. coli*, PCV2, *L. intracellularis*

## Abstract

Efficient, high-quality production is essential to the swine industry, with carcass inspection by health authorities playing a crucial role in detecting abnormalities that may lead to condemnation, including enteritis. Enteritis is, usually, characterized by changes in color, mucosal integrity, intestinal wall thickness and luminal contents and has a multifactorial etiology. This study assessed and classified visual lesions attributed to enteritis and to seek for associations with known pathogens. A scoring system was applied to classify visual changes, and 30 condemned intestines were categorized as mild (< 10 points), moderate (11–20 points), or severe (> 20 points). Laboratory analyses included histopathology; isolation and antimicrobial susceptibility testing of *Escherichia coli* and *Salmonella* spp.; and molecular detection of porcine circovirus type 2 (PCV2), *Lawsonia intracellularis*, *Brachyspira pilosicoli*, and *Brachyspira hyodysenteriae*. Lesions were classified as mild (23%), moderate (57%), and severe (20%). PCV2 and *L. intracellularis* DNA were detected in 60% and 23% of samples, respectively. *E. coli* was isolated from 57% of intestines, of which 87% exhibited antimicrobial resistance and 56% were multidrug-resistant. No samples tested positive for *Salmonella* spp., *B. pilosicoli*, or *B. hyodysenteriae*. Lower condemnation scores were associated with the presence of PCV2 DNA, mild lesions, and lymph nodes showing necrosis and follicular depletion. Mild lesions were also associated with a lower frequency of multidrug-resistant *E. coli*. The high prevalence of antimicrobial resistance is concerning from a One Health perspective. These findings underscore the importance of pathogen monitoring, antimicrobial stewardship, and strong animal welfare practices within the swine production chain.

**Supplementary Information:**

The online version contains supplementary material available at 10.1007/s11259-026-11200-9.

## Introduction

The pork production stands out in Brazil and is expanding, with an annual slaughter in 2023 of 57.17 million animals, 5.29 million tons of pork meat, of which 1.23 million were exported (ABPA 2024). In Brazil, the safety of animal origin foods is achieved by a system that includes veterinarians belonging to the Health Authorities that are responsible for meat inspection (de Freitas Costa et al. 2020). Another important issue related to foodborne pathogens is antimicrobial resistance (AMR). It is known that animal production utilizes antimicrobials (sometimes substances overlap broadly between human and animal medicine) as a tool in preventing treatment or growth promoter. Thus, similar resistant microorganisms might spread among humans, animals and the environment.

In the slaughterhouse, carcasses (or part of them) identified as unfit for human consumption are removed from the food chain, causing losses around US$ 28 million per year to the Brazilian pork industry (Machado et al. 2018). This approach has allowed over the years to keep the previously most common zoonoses under control, including tuberculosis, cysticercosis, trichinellosis, and others (Coldebella et al. 2017; Kich 2019).

Enteric diseases in grower-finisher pigs are present around the world and can be caused by many agents, like *Lawsonia intracellularis, Brachyspira* spp.*, Escherichia coli, Salmonella* spp. and *Porcine Circovirus* type 2 (Luppi et al. 2023). The condemnation of the carcass or organs comprises visual-only inspections (VOI) before and after slaughtering, following the quality control program of the industry, when being audited by the Health Authorities (Kich 2019). However, the visual appearance of the carcass or offal does not necessarily indicate etiology, nor if it is caused by a zoonotic pathogen. In fact, there are no studies about lesions classification or presence of pathogens in condemned carcasses after VOI implementation. Therefore, the aim of this study was to classify visual intestine lesions attributed to enteritis and to seek for associations with known pathogens.

## Material and methods

The study took place from June to December 2023 at a pig slaughterhouse under federal inspection in Rio Grande do Sul, Brazil. Intestines included in this study were selected based on their classification as enteritis by visual-only inspection (MAPA 2018). This classification was established after pathological evaluation and final diagnosis by the official veterinary inspector, following the slaughterhouse’s Standard Operational Procedure manual, which is approved by the corporate quality department and the official inspection authorities. Over this period, the slaughterhouse was visited sixteen times, and a total of 30 intestines and 27 corresponding mesenteric lymph nodes were collected.

To evaluate the visual-only *postmortem* intestine lesions, a severity scoring system based on specific pathological features was defined. Each affected area was photographed for posterior visual analyses and comparison between samples (Supplementary file 2). A total of fourteen characteristics, including lymph nodes reactivity, were evaluated and classified individually as a score of 0 and 3 according to the visual lesion severity (Table S1). The gap of the summatory of these fourteen lesions is zero to forty-two, and the sample is classified as mild (up to 10 points), moderate (11–20 points) or severe (more than 20 points).

For *Salmonella* spp. detection, the samples were processed by bacterial isolation according to International Organization for Standardization (ISO) 6579:2017. For *Escherichia coli*, the samples were processed by conventional bacterial isolation according to Normative Instruction n. 62 (MAPA 2003). All isolates were also screened for the presence of *F18*, *F41*, *F4*, F5 fimbriae genes and *StB*, *LtB*, *StaP*, *Stx2*, *987P* toxins genes by PCR according to CLSI 9107, using a commercial kit (IndiMag Pathogen Kit, Indical Bioscience, Germany; NewGene BPAmp, Simbios Biotechnology, Brazil). The isolates were subjected to antimicrobial susceptibility test against ten different antimicrobial agents using disk-diffusion tests: amoxicillin (10 µg); ceftiofur (30 µg); ciprofloxacin (10 µg); gentamicin (10 µg); lincomycin + spectinomycin (109 µg); neomycin (10 µg); norfloxacin (30 µg); sulfa + trimethoprim (25 µg) and enrofloxacin (5 µg), according to CLSI 2018. For colistin, the Minimal Inhibitory Concentration (MIC) was determined, according to Andrews (2001).

For detection of *Brachyspira pilosicoli*, *Brachyspira hyodysenteriae*, *Lawsonia intracellularis*, and Porcine Circovirus type 2, DNA was extracted from intestines and mesenteric lymph nodes. For pathogens’ detection, commercially available PCR were used (IndiMag Pathogen Kit, Indical Bioscience, Germany; NewGene, Simbios Biotechnology, Brazil). Formalin fixed paraffin embedded samples of intestines and mesenteric lymph nodes were examined by hematoxylin and eosin.

Either chi-square test or Fisher's exact test were applied to search for association variables examined with two key outcomes: (i) carcass condemnation rates based on intestine lesions, and (ii) classification of visual lesions (Tables 1 and 2). Statistical significance was set at *p* ≤ 0.05.

**Table 1:** Condemnation frequency over the independent variables analyzed. Chi-square association test or Fisher exact test were performed

Variable	Category	Samples (*n*)	Condemnation *n* (%)	*P* value
PCV2	Presence	18	11 (61)*	0.014*
	Absence	12	12 (100)	
Visual -only score lesion (intestines)	Mild	7	3 (42)*	0.050*
	Moderate	17	14 (82)	
	Severe	6	6 (100)	
Multidrug resistance	Presence	7	4 (57)	0.063
	Absence	9	9 (100)	
*E coli*	Presence	17	14 (82)	0.403
	Absence	12	8 (66)	
*Lawsonia* spp.	Presence	4	4 (100)	0.548
	Absence	26	19 (73)	
AMR	Presence	14	12 (85)	0.350
	Absence	2	1 (50)	
Lymph nodes	Other causes	6	5 (83)	0.022*
	No alteration	13	12 (92)*	
	Necrosis/follicular depletion	8	3 (37)*	

^*^p ≤ 0.05

**Table 2:** Visual-only score of intestines condemned in slaughterhouse over the independent variables analyzed

Variable	Category	Samples (*n*)	Intestinal visual classification *n* (/%)			*P* value
			Mild	Moderate	Severe	
AMR	Yes	16	2 (12)	9 (56)	3 (19)	0.740
	No	2	2 (100)	0 (0)	0 (0)	
PCV2	Presence	18	5 (28)	10 (55)	3 (17)	0.781
	Absence	12	2 (17)	7 (58)	3 (25)	
Multidrug resistance	Yes	9	0 (0)*	6 (67)	3 (33)	0.015*
	No	7	4 (57)	3 (43)	0 (0)	
*E coli*	Presence	17	4 (57)	10 (59)	3 (18)	1.000
	Absence	12	2 (17)	7 (58)	3 (25)	
*Lawsonia* spp.	Presence	4	0 (0)	2 (50)	2 (50)	0.184
	Absence	26	7 (27)	15 (58)	4 (15)	
Carcass Condemnation	Yes	23	3 (13)*	14 (61)	6 (26)	0.050*
	No	7	4 (57)	3 (43)	0 (0)	
Lymph nodes	Other causes	6	1	4	1	0.553
	No alteration	13	3	6	4	
	Necrosis/follicular depletion	8	2	6	0	

^*^p ≤ 0.05

## Results

Of the pig carcasses evaluated, 23 (77%) were condemned due to intestinal lesions. In the macroscopic evaluation of the 30 intestines, 17 (57%) had moderate, 6 (20%) mild, and 7 (23%) had severe lesions (Table S2; Figure S1). The common histological changes observed were lymphoplasmacytic enteritis (43%), necrotic enterocolitis (20%), chronic serositis (17%) and parasitic enteritis (7%). With respect to lymph nodes alterations, the common histological changes observed were necrosis and follicular depletion.

**Fig. 1:**
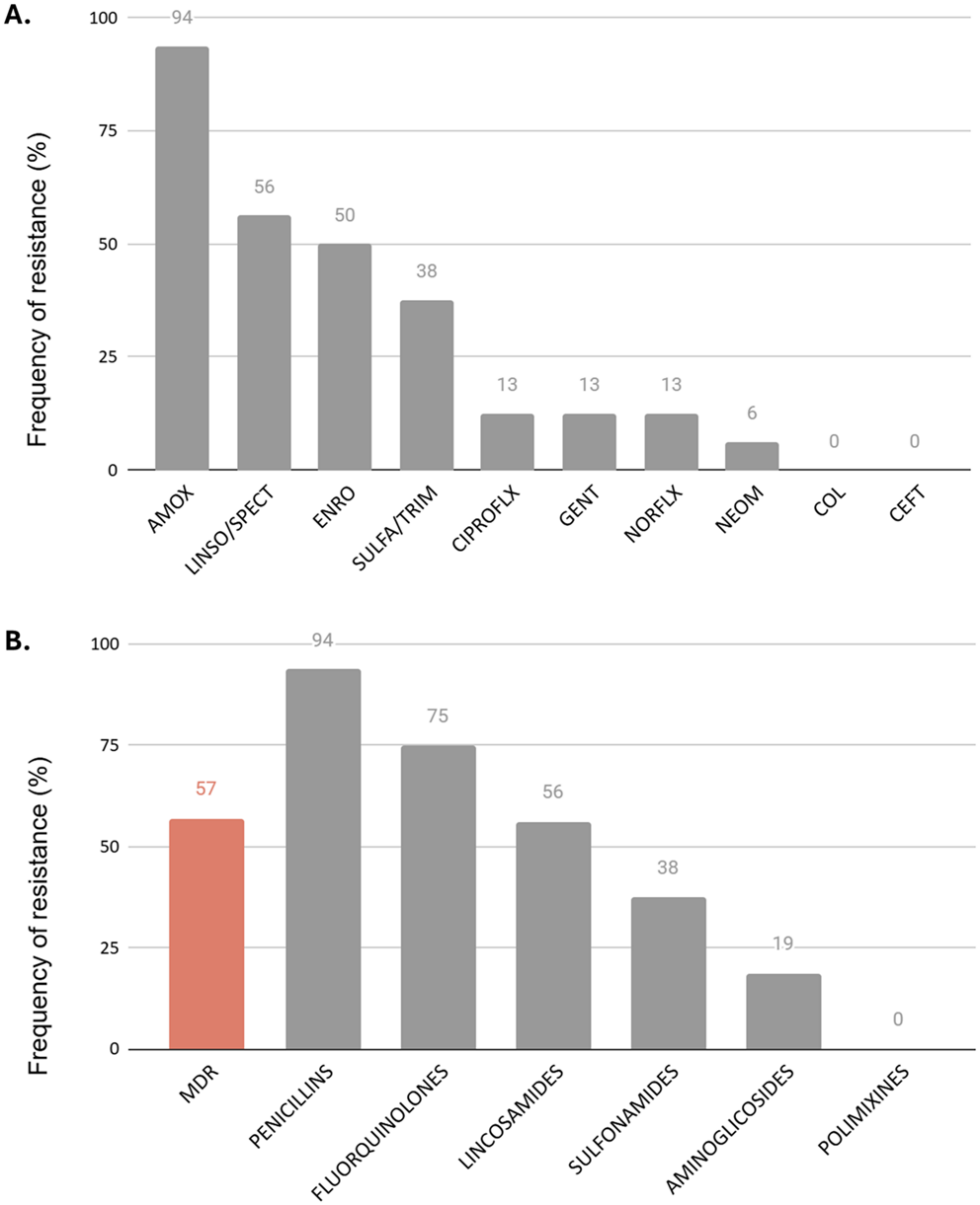
**A** Frequency of non-hemolytic *Escherichia coli* isolated from intestines with enteritis, according to the antimicrobial agent; **B** Frequency of non-hemolytic *Escherichia coli* isolated from intestines with enteritis, according to the antimicrobial classes. AMX amoxicillin; CIP ciprofloxacin; CFT ceftiofur; COL colistin; ENO enrofloxacin; GEN gentamicin; L lincomycin; NEO neomycin; NOR norfloxacin; SXT sulfamethoxazole + trimethoprim

Non-hemolytic *Escherichia coli* was isolated in 17 (57%) of the samples. Of these isolates, 93% were susceptible to amoxicillin, 87% presented antimicrobial resistance (AMR), and 57% were considered multidrug-resistant (MDR) (resistant to 3 or more antimicrobial classes). Of antimicrobial classes, isolates were shown to be more susceptible to penicillins, followed by fluoroquinolones and lincosamides (Figs. 1 A and B). Two isolates had fimbriae and toxins genes detected: *StaP* and *Stx2e*; and *F18* and *Stx2e*. None of the samples had *Salmonella* isolated.

Regarding molecular analyses, 18 (60%) samples had PCV2 DNA detected. *Lawsonia intracellularis* was detected in 4 (23%) samples. None of the samples had observed pathognomonic lesions of *Brachyspira pilosicoli* and *Brachyspira hyodysenteriae* nor their DNA detected.

In statistical analysis, lower condemnation rates were observed in lesions classified as mild (Table 1). Carcasses from animals with detectable PCV2 DNA had a lower likelihood of being condemned. Additionally, microscopic lesions in lymph nodes were linked to a higher rate of carcasses condemnation. The only factor related to visual-only score of intestines was multidrug resistance: intestines with mild lesions showed lower-than-expected levels of multidrug resistance (Table 2).

## Discussion

The lesion scoring system developed in this study proved to be appropriate, as milder lesions were correlated with a lower likelihood of carcass condemnation. Moreover, there was a negative correlation between PCV2 DNA detection and carcass condemnation. PCV2 is an important agent commonly found in co-infections in swine, which contributes to development of necrotic enteritis lesions (Opriessnig et al. 2011). Nevertheless, PCV2 infection is frequently associated with subclinical manifestations, particularly in vaccinated herds, which is the case in the population evaluated in this study. However, further studies are warranted to better elucidate the relationship between PCV2 detection and carcass condemnation outcomes.

Carcasses with unaltered lymph nodes were more likely to be condemned due to intestinal lesions indicating that enteritis-related intestinal condemnation does not necessarily involve lymph node alterations. All lymph node samples with discrete necrosis/depletion were PCV2 positive in molecular analyses, but not all the PCV2 positives had lymph node affections. A limitation of this study is that sample collection was based on the presence of visible lesions on the slaughter line; therefore, the collected intestinal segments did not always include Peyer’s patches, which prevented confirmation of the diagnosis of PCV2 enteric disease (Segalés, 2012). Moreover, although a single trained evaluator assessed the viscera in situ to ensure a more consistent and reliable classification, the available photographs were obtained without a standardized imaging protocol. Thus, future studies adopting standardized photographic documentation are needed to facilitate the application and reproducibility of the scoring system and to further validate this protocol.

Regarding the histopathological alterations detected, they are usually related to infections such as PCV2 and *L. intracellularis* (lymphoplasmacytic enteritis; Opriessnig et al. 2011), *Glaesserella parasuis* (chronic serositis; Salogni et al. 2020), *Salmonella* spp., *Escherichia coli, Brachyspira hyodisenteriae* (necrotic enterocolitis; Luppi et al. 2023), and *Cystoisospora suis* (parasitic enteritis; Nunes et al. 2023). However, due to the low number of samples, we could not perform a correlation analysis. Moreover, not all the pathogens mentioned were accessed in this study and may be subject for further studies in a higher number of samples.

The results revealed a high level of antimicrobial resistance among non-hemolytic *E. coli* isolates across several antibiotic classes. Although these isolates are likely commensal and not pathogenic to swine, their resistance profiles are concerning because many of the affected antimicrobials, such as amoxicillin and lincomycin, are widely used in human medicine. Reflecting these concerns, several antibiotics have been prohibited as performance-enhancing feed additives in Brazilian swine production, including colistin sulfate, lincomycin, tiamulin, and tylosin (MAPA 2016; MAPA 2020). Nevertheless, lincomycin exhibited the second-highest resistance rate in this study. Other Brazilian studies have also reported high levels of resistance to amoxicillin (Dutra et al. 2021; Oliveira et al. 2024), highlighting the influence of this drug in both veterinary and human healthcare for treating common infections. Despite the limited number of samples, overall, the AMR patterns observed here are consistent with findings reported worldwide (Van Thong et al. 2024; Bassitta et al. 2022; Bassi et al. 2023), reflecting the widespread dissemination of antimicrobial resistance among enteric pathogens in swine production systems.

In the present study, intestinal lesions classified as mild showed a lower frequency of multidrug resistance. A few aspects could be considered to explain these results such as the lower use of antimicrobials in animals with mild lesions, leading to a relaxed resistant bacterial selection compared to the animals with more severe lesions. However, this hypothesis must be tested in future studies, since we had no access to the antimicrobial use record of evaluated animals.

The etiological agents’ variability and the AMR profile demonstrate the repercussions of the swine farmers management. Although the world’s pression for reducing the use of antibiotics, in Brazil the use is still considerate indispensable, legitimate and beneficial. According to Albernaz-Gonçalves et al. (2021), it is difficult to change deep-rooted habits of the farmers. Low adoption of biosecurity and hygiene measures also are allied to the excessive use of antibiotics. The correct adoption of these practices may help to reduce the use of antibiotics and, consequently, the emergence of multidrug-resistant bacteria.

It is important to point out that this study performed a correlation analysis, which does not imply causality between the observed variables. Studies with different designs such as a case x control may help to explain the observed data. Moreover, other limitations include the low number of evaluated animals, the limited diagnosis applied, precluding identifying other etiological agents, the lack of access to information regarding animals’ clinical information, management and antibiotic exposure.

## Conclusions

The study results suggest practical limitations in using a lesion classification model for enteritis due to the numerous macroscopic findings in the gut, which may or may not be associated with enteric disorders. However, since mild lesions were correlated with lower condemnations, the score system developed could be validated in a higher number of samples, and other slaughterhouses as a tool to guide condemnation based on VOI. Nonetheless, although with a limited number of samples evaluated, the antibiotic resistance profile highlights the need for prudent antibiotic use in pork production, emphasizing not only a reduction in antibiotic applications per pig but also improvements in biosecurity and hygiene practices among farmers.

## Supplementary Information

Below is the link to the electronic supplementary material.

Supplementary file1 (ODT 3.37 MB)

Supplementary file2 (PDF 13.3 MB)

## Data Availability

The datasets generated during and/or analyzed during the current study are available from the corresponding author on reasonable request.
