# Supplementary material for: Visual and microscopic lesions of enteritis in slaughtered swine: pathogen identification and antimicrobial resistance implications
Source: Vet Res Commun. 2026 Apr 27;50(4):288. doi: 10.1007/s11259-026-11200-9 (PMC13121305; doi:10.1007/s11259-026-11200-9)
Supplement: Supplementary file 2 — (PDF 13.3 MB) [file 11259_2026_11200_MOESM2_ESM.pdf]

Supplementary file 1. Photographs of intestines evaluated in the present study.

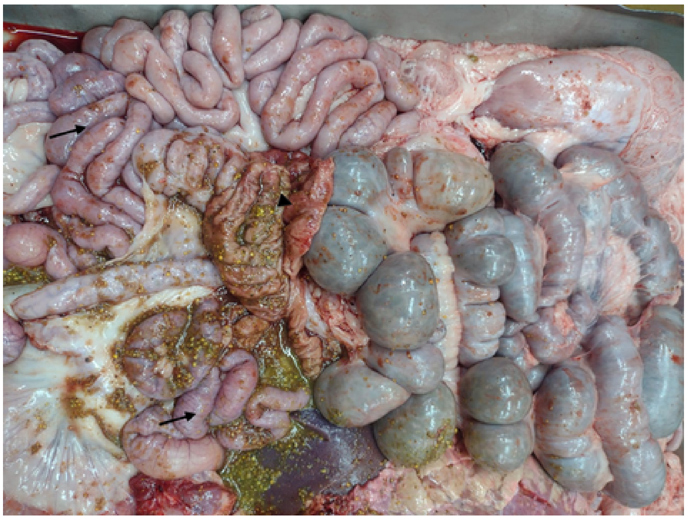

Sample 1. Swine. Segment of small intestine with mild serosal hyperemia (arrow). The mucosa surface shows diffuse, mild hyperemia and hemorrhage (arrowhead).

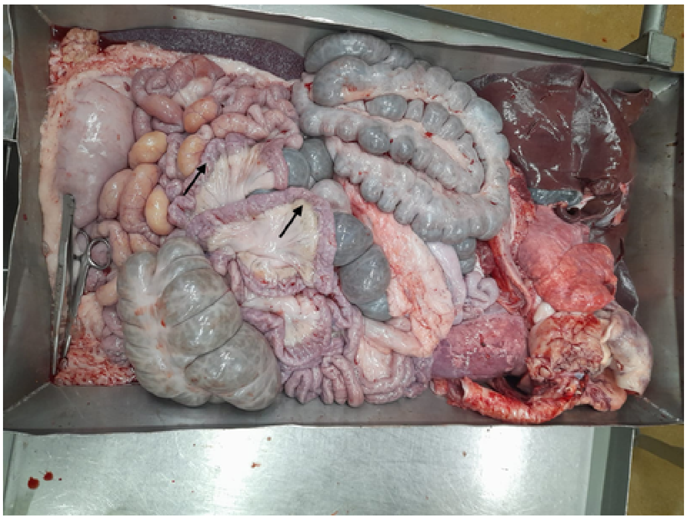

Sample 2. Swine. Segment of small intestine with moderate wall thickening (arrow), with serosal and mesenteric edema and multifocal hemorrhage (arrowhead).

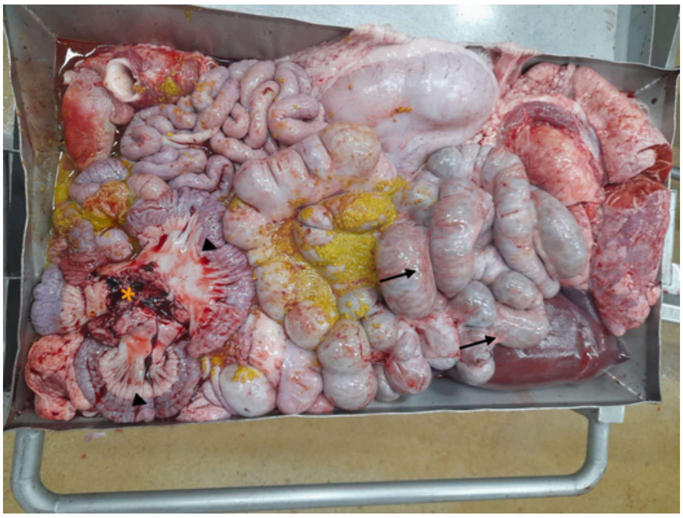

Sample 3. Swine. Segment of large intestine with a small, fibrillar deposit on the serosal surface (serositis; arrow). Segment of small intestine with moderate wall thickening and moderate serosal hyperemia (arrowhead). The mesentery presents focal hemorrhage (*).

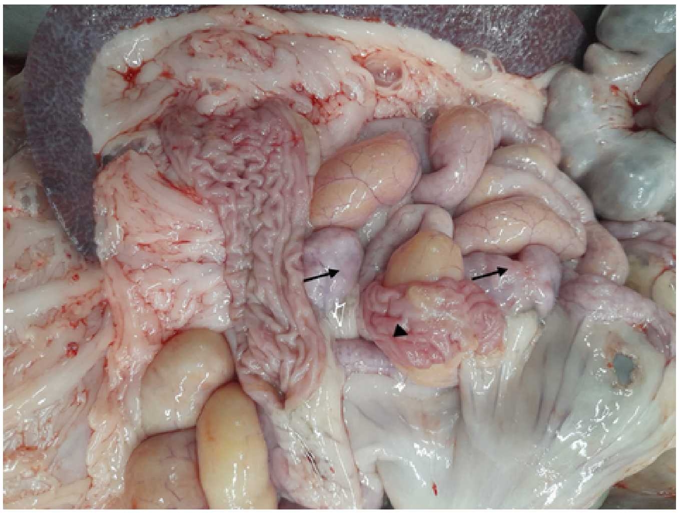

Sample 4. Segment of small intestine with milld wall thickening with serosal edema (arrow).The mucosal surface shows mild hyperemia (arrowhead).

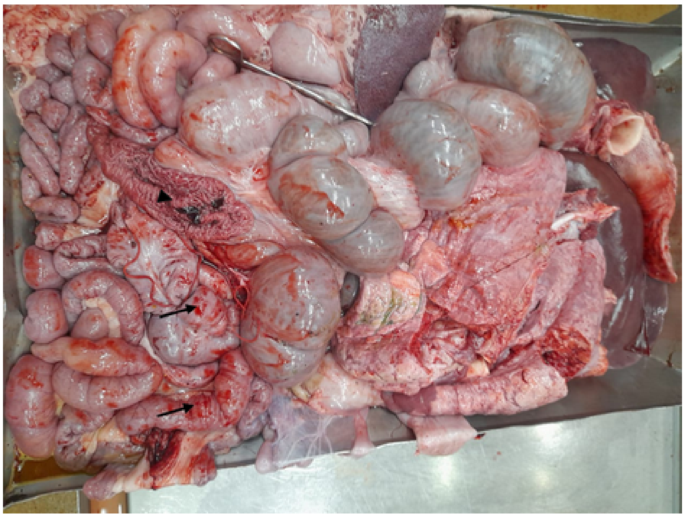

Sample 5. Swine. Segment of small intestine with diffuse, moderate serosal hyperemia and multifocal hemorrhage (arrow). The mucosa surface shows diffuse, moderate hyperemia and hemorrhage (arrowhead).

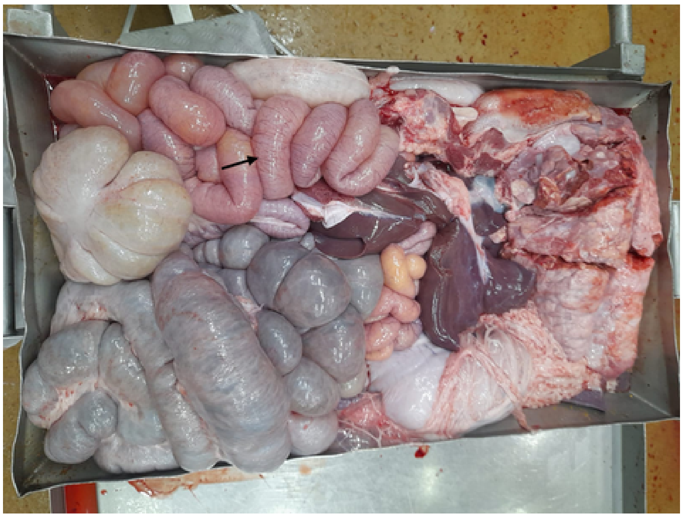

Sample 6. Swine. Segment of small intestine with diffuse, mild serosal hyperemia and marked luminal dilatation (arrow). The colon shows mild serositis (arrowhead).

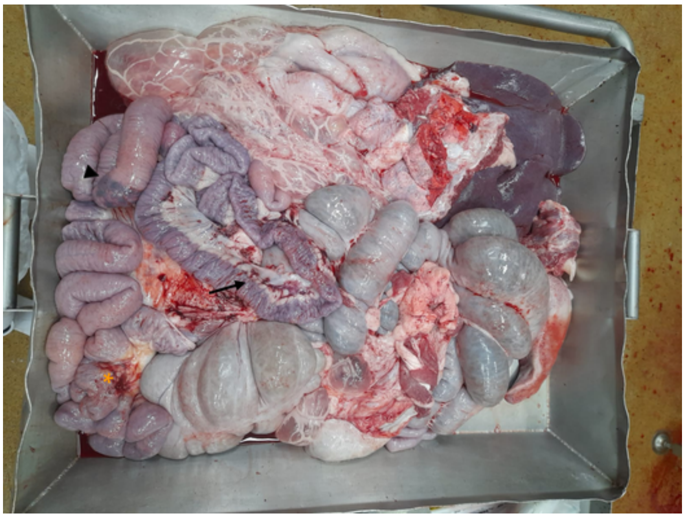

Sample 7. Swine. Segment of small intestine with moderate serosal congestion (arrow). Areas of moderate luminal dilation, thinning of the intestinal wall, and apparently serosanguineous contents are observed (arrowhead). The mesentery and omentum show moderate edema and focal hemorrhage (*).

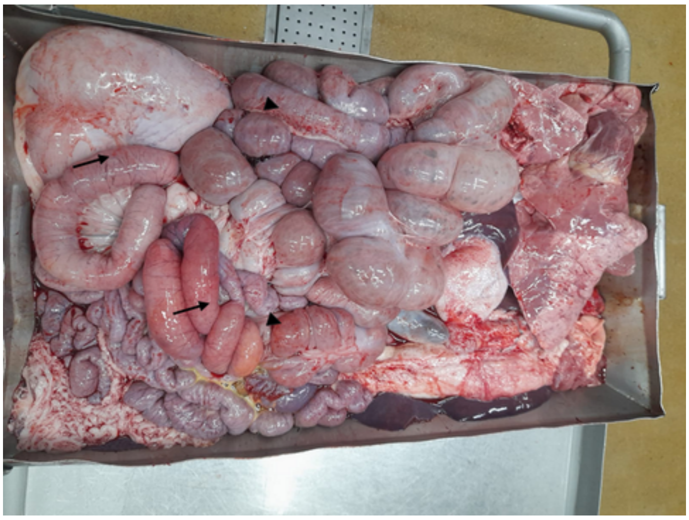

Sample 8. Swine. Small and large intestines with moderate serosal hyperemia (arrow) and multifocal hemorrhage (arrowhead).

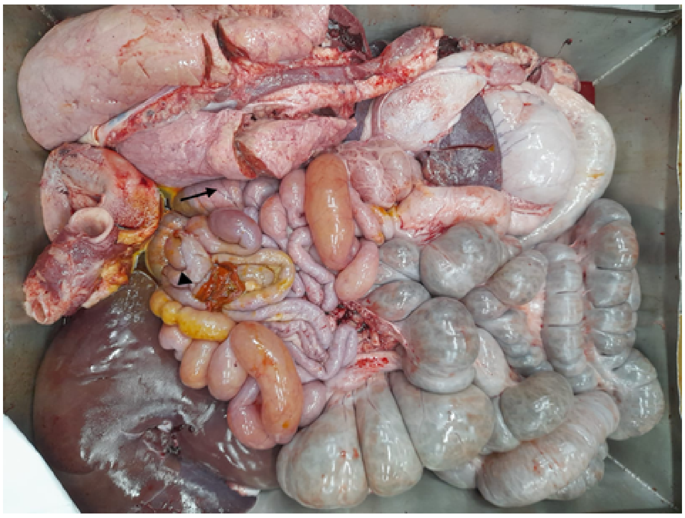

Sample 9. Swine. Segment of small intestine with mild serosal hyperemia (arrow). The mucosa surface shows diffuse, moderate hyperemia and hemorrhage (arrowhead).

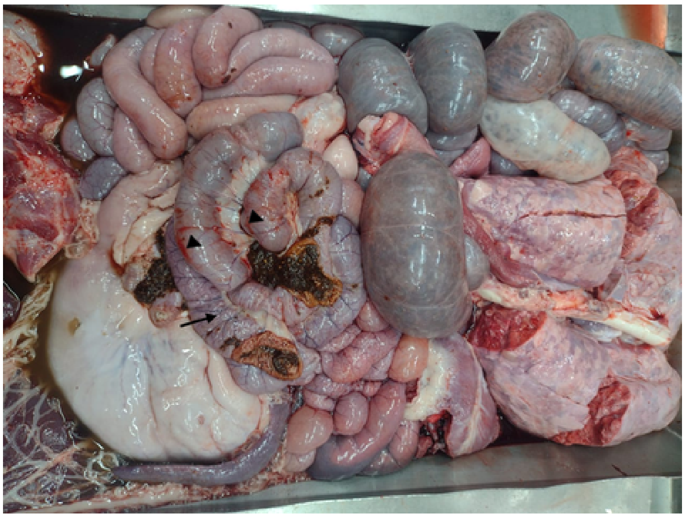

Sample 10. Swine. Segment of small intestine moderate serosal congestion (arrow) and multifocal hemorrhage (arrowhead).

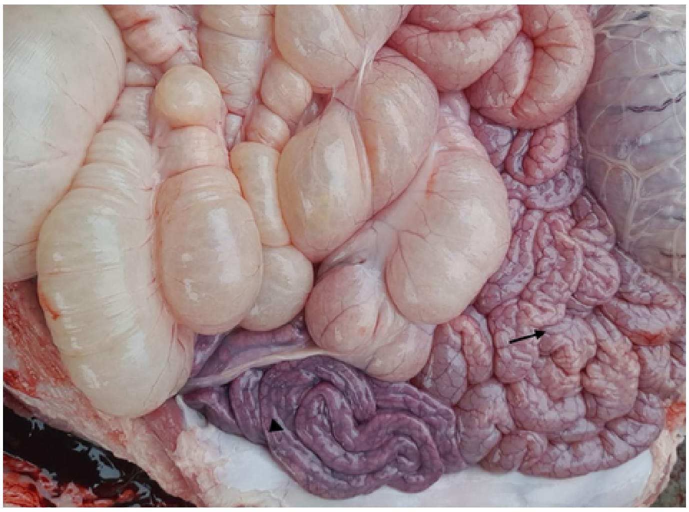

Sample 11. Swine. Small intestine with segments of moderate (arrow) to marked serosal congestion (arrowhead).

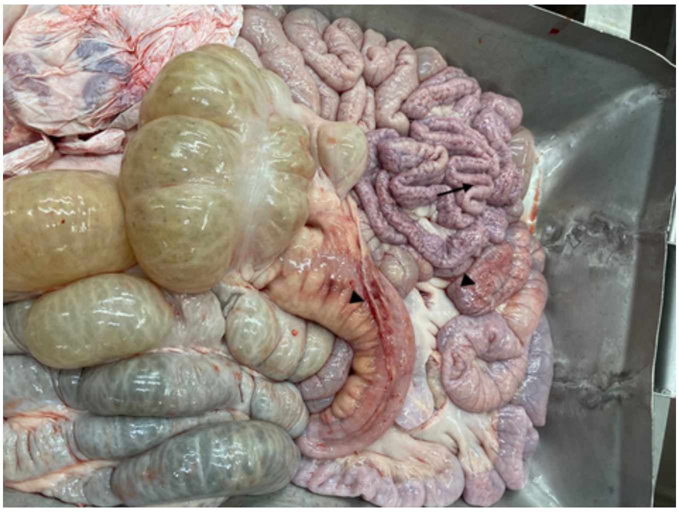

Sample 12. Swine. Segment of small intestine with multifocal, moderate serositis (arrow).

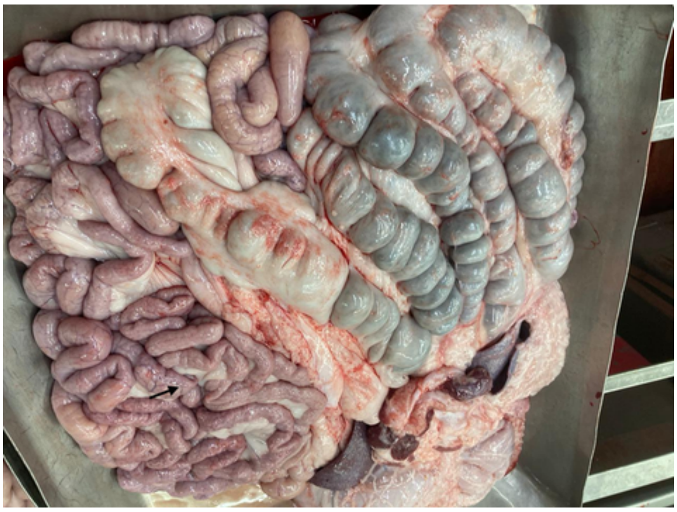

Sample 13. Swine. Segment of small intestine with small wall thickening and moderate serosal hyperemia (arrow). The cecum present moderate serositis (arrowhead).

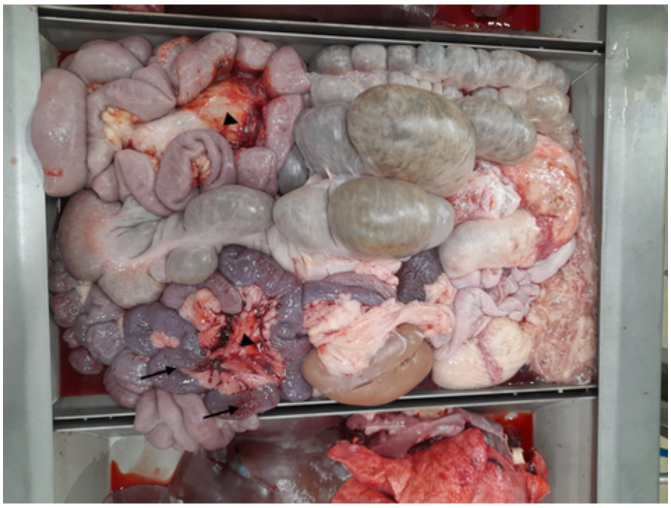

Sample 14. Swine. Segment of small intestine with marked serosal congestion and multifocal hemorraghe (arrow). The mesentery and mesocolon present hemorrhage and edema (arrowhead).

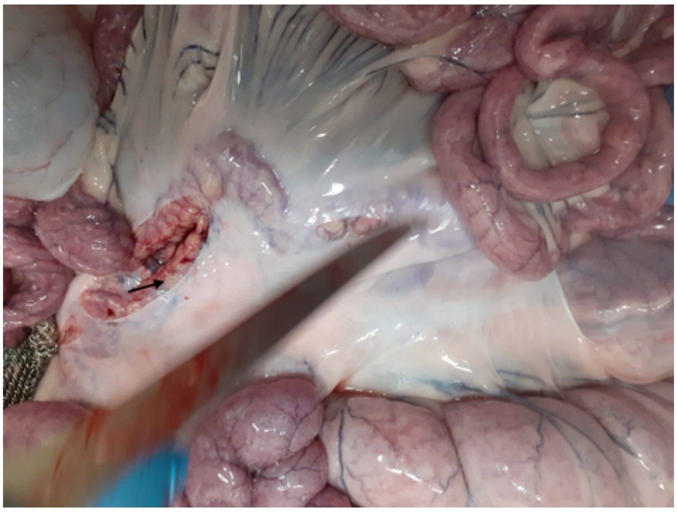

Sample 15. Swine. Mesenteric lymph nodes moderately enlarged (arrow).

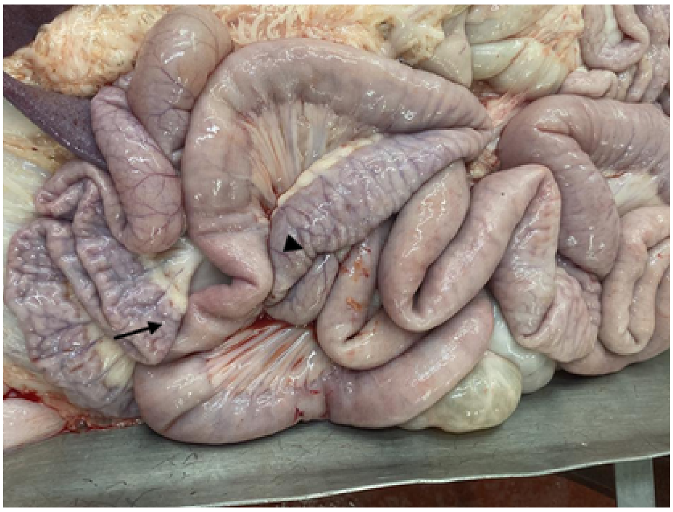

Sample 16. Swine. Segment of small intestine with moderate wall thickening (arrow) and moderate serosal congestion and edema (arrowhead).

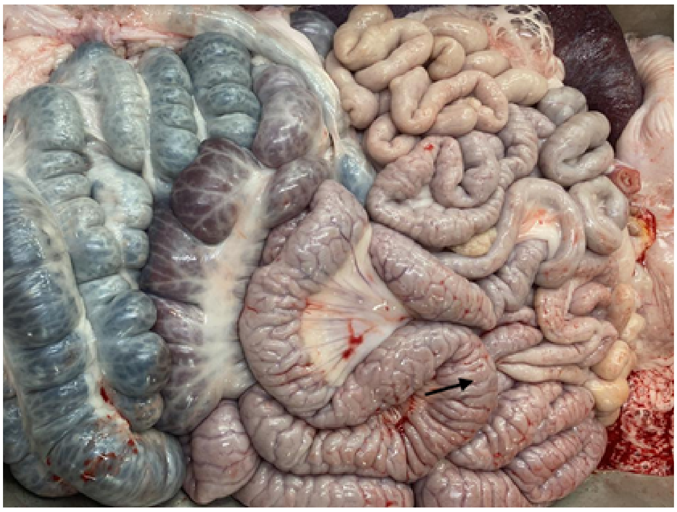

Sample 17. Swine. Segment of the small intestine with diffusely thickened and corrugated serosa (arrow).

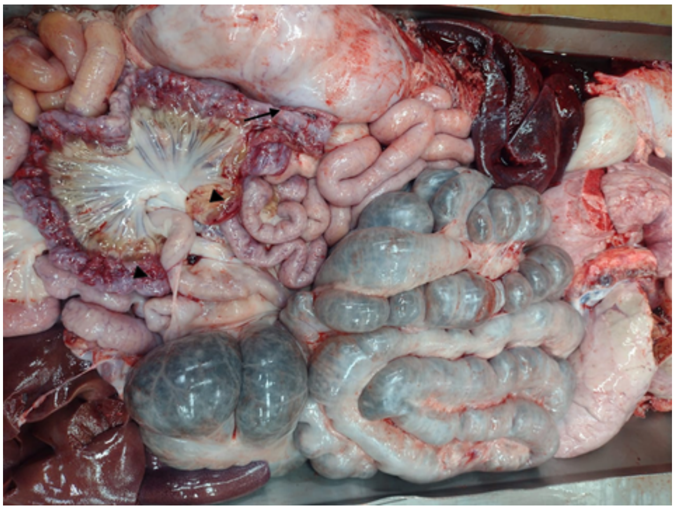

Sample 18. Swine. Intestinal segments with moderate serositis (arrow). Mesentery with edema and hemorrhage (arrowhead).

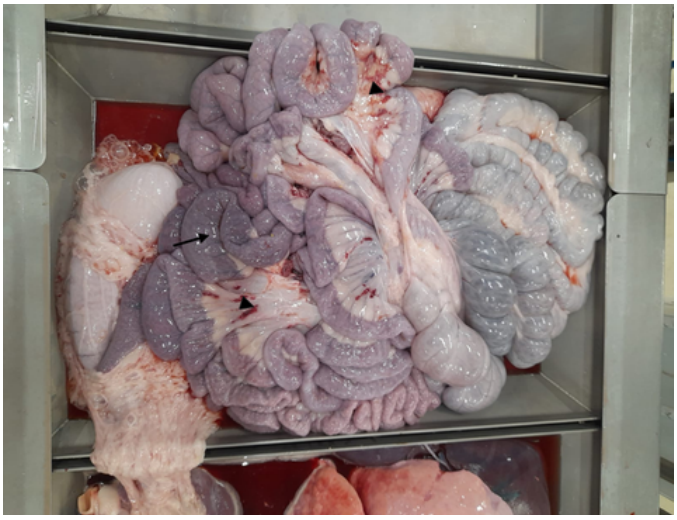

Sample 19. Swine. Segment of small intestine with moderate serosal congestion(arrow). Mesentery and serosa with areas of edema and multifocal hemorrhage (arrowhead).

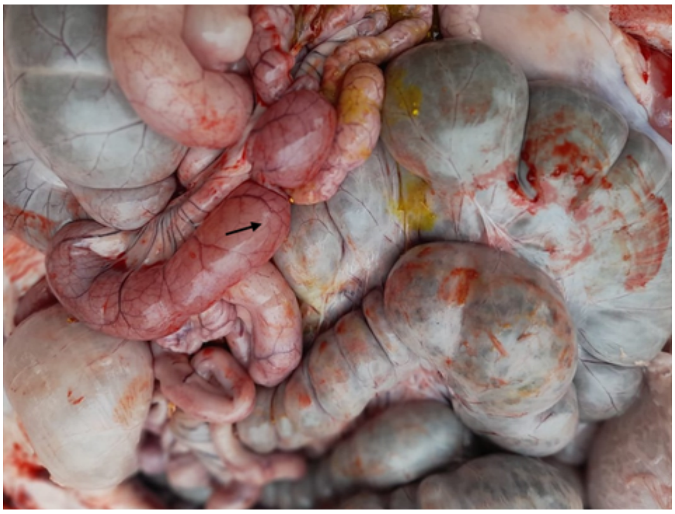

Sample 20. Swine. Segment of small intestine with focal, serosal hyperemia and marked luminal dilatation.

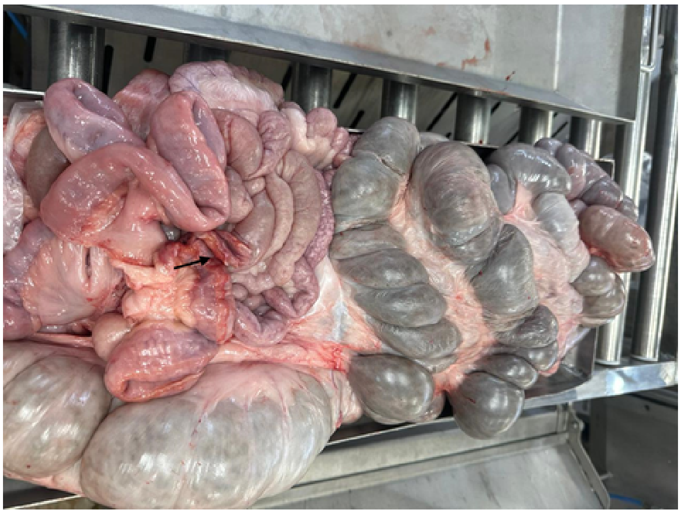

Sample 21. Swine. Segment of small intestine with moderate serositis (arrow).

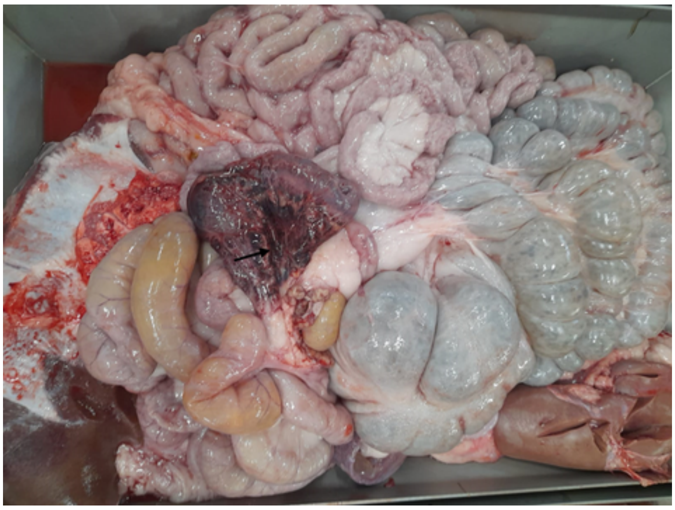

Sample 22. Swine. Segment of small intestine with intense serosal hemorrhage, edema andnecrosis with extension to the adjacent mesentery (arrow).

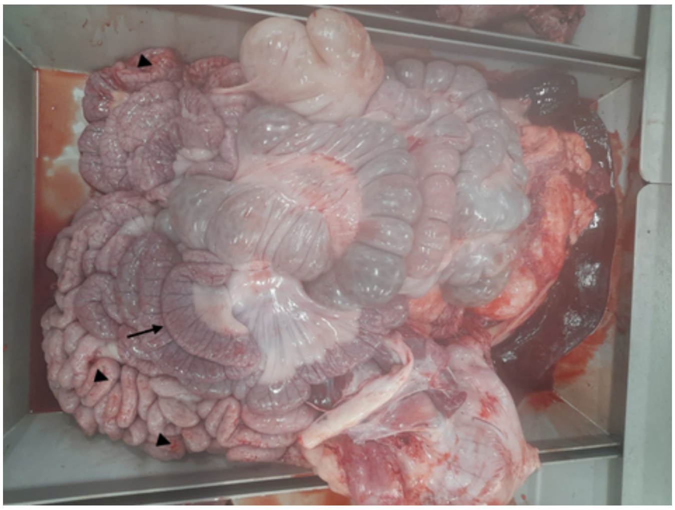

Sample 23. Swine. Segment of small intestine with diffuse, moderate serosal congestion (arrow) and multifocal hemorrhage (arrowhead).

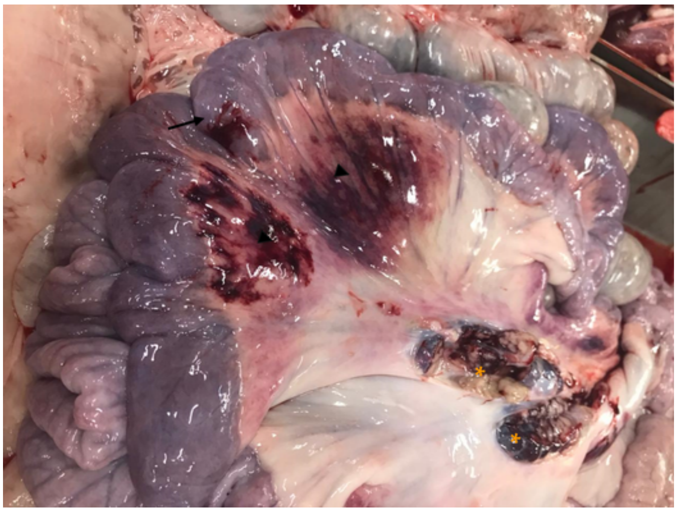

Sample 24. Swine. Segment of small intestine with diffuse, intense wall congestion (arrow) with areas of necrosis and hemorrhage extending into the adjacent mesentery (arrowhead). Mesenteric lymph nodes moderately enlarged (*).

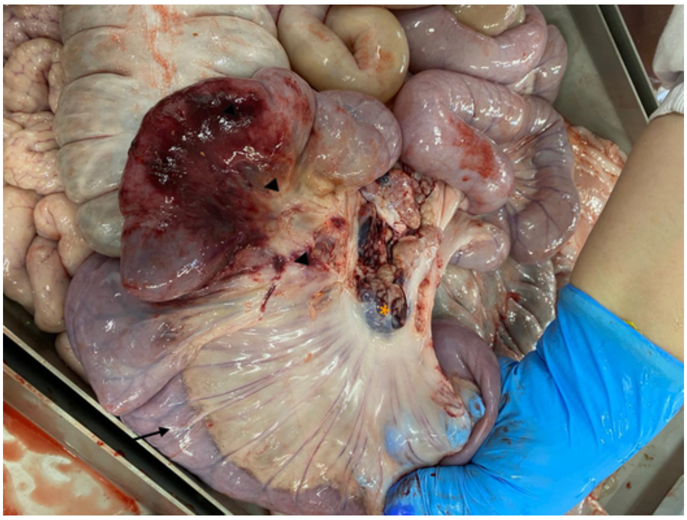

Sample 25. Swine. Segment of small intestine with diffuse, moderateserosal hiperemy (arrow) with area of necrosis and hemorrhage extending into the adjacent mesentery (arrowhead). Mesenteric lymph nodes moderately enlarged (*).

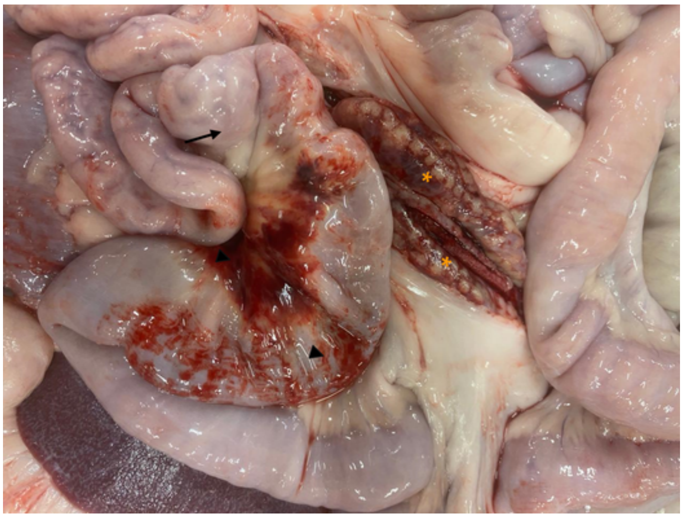

Sample 26. Swine. Segment of small intestine with moderate wall thickening (arrow) and moderate serosal hemorrhage extending into the adjacent mesentery (arrowhead). Mesenteric lymph nodes intensively enlarged (*).

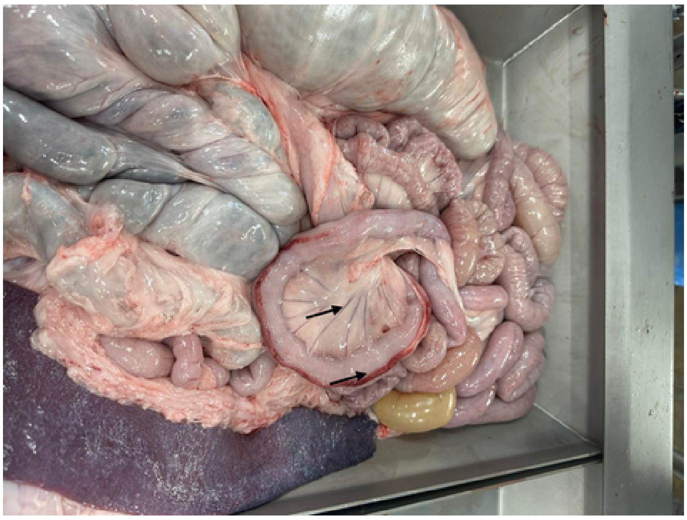

Sample 28. Swine. Segment of small intestine with a focal area of wall hyperemia and serosal edema (arrow).

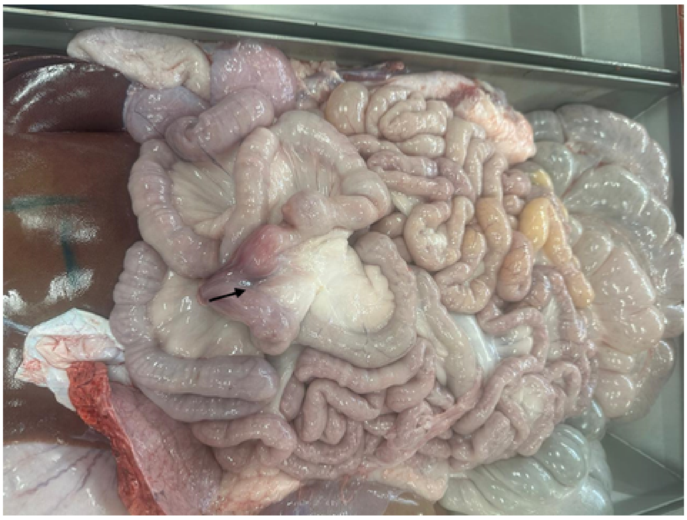

Sample 27. Swine. Segment of small intestine with mild serosal and mesenteric hyperemia (arrow).

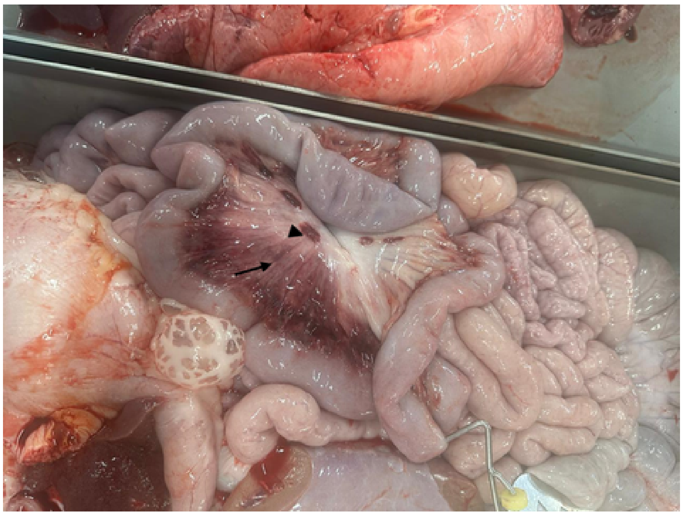

Sample 29. Swine. Segment of small intestine with an area of hemorrhage and edema in the serosa and mesentery (arrow). Mesenteric lymph nodes are hyperemic (arrowhead).

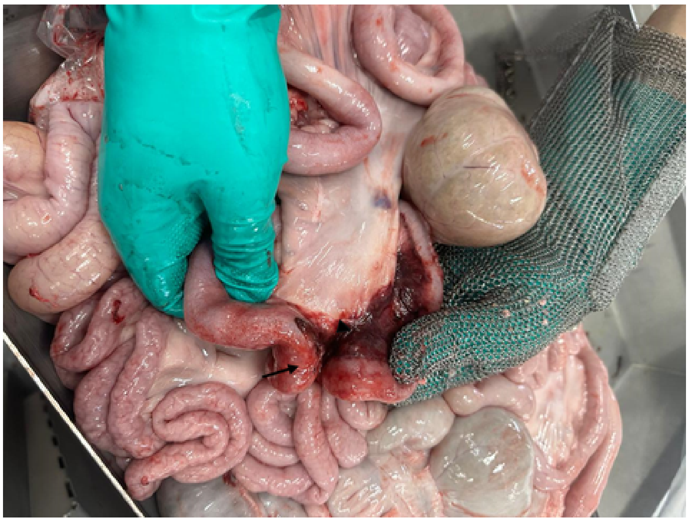

Sample 30. Swine. Segment of small intestine with diffuse, moderate serosal hyperemia with necrosis and hemorrhage extending into the adjacent mesentery (arrow).
