# Supplementary material for: Visual and microscopic lesions of enteritis in slaughtered swine: pathogen identification and antimicrobial resistance implications
Source: Vet Res Commun. 2026 Apr 27;50(4):288. doi: 10.1007/s11259-026-11200-9 (PMC13121305; doi:10.1007/s11259-026-11200-9)
Supplement: Supplementary file 3 [file 11259_2026_11200_MOESM1_ESM.odt]

Supplementary tables

Table S1.Classification according to severity of visual -only inspection of intestine lesions. The classification is given by the sum of the affections, being Mild (0 to 10 points), Moderate (11 to 20 points) or Severe (more than 21 points).

| Sample | Hyperemia | Hemorragy | Edema | Necrosis | Extension | Serositis | Abscess | Lymph Nodes | Dilatation/gas | Exudate | Transudate | Carcass repercussion | Omentum thickening | Other organs | Sum | Classification |
| --- | --- | --- | --- | --- | --- | --- | --- | --- | --- | --- | --- | --- | --- | --- | --- | --- |
| 1 | 1 | 1 | 1 | 0 | 0 | 0 | 0 | 1 | 0 | 0 | 0 | 1 | 1 | 0 | 6 | Mild |
| 2 | 1 | 1 | 3 | 0 | 2 | 2 | 0 | 2 | 1 | 0 | 2 | 1 | 1 | 1 | 17 | Moderate |
| 3 | 2 | 3 | 1 | 1 | 0 | 0 | 0 | 1 | 1 | 0 | 0 | 2 | 0 | 0 | 11 | Moderate |
| 4 | 1 | 1 | 2 | 0 | 2 | 1 | 0 | 2 | 2 | 0 | 1 | 1 | 1 | 1 | 15 | Moderate |
| 5 | 2 | 2 | 1 | 0 | 2 | 1 | 0 | 2 | 1 | 0 | 1 | 1 | 1 | 1 | 15 | Moderate |
| 6 | 1 | 0 | 2 | 0 | 1 | 1 | 0 | 1 | 3 | 0 | 1 | 1 | 1 | 1 | 13 | Moderate |
| 7 | 2 | 1 | 2 | 1 | 1 | 1 | 0 | 2 | 2 | 0 | 2 | 1 | 1 | 1 | 17 | Moderate |
| 8 | 2 | 2 | 2 | 1 | 3 | 1 | 0 | 3 | 2 | 0 | 2 | 2 | 2 | 2 | 24 | Severe |
| 9 | 1 | 2 | 0 | 0 | 0 | 1 | 0 | 1 | 1 | 0 | 1 | 0 | 1 | 1 | 9 | Mild |
| 10 | 1 | 3 | 0 | 0 | 1 | 0 | 0 | 1 | 1 | 0 | 0 | 3 | 0 | 1 | 11 | Moderate |
| 11 | 3 | 1 | 1 | 0 | 0 | 0 | 0 | 1 | 0 | 0 | 0 | 2 | 0 | 0 | 8 | Mild |
| 12 | 1 | 2 | 1 | 0 | 2 | 2 | 0 | 1 | 2 | 3 | 3 | 2 | 3 | 1 | 23 | Severe |
| 13 | 2 | 2 | 1 | 0 | 2 | 2 | 0 | 2 | 1 | 0 | 0 | 0 | 1 | 0 | 13 | Moderate |
| 14 | 2 | 2 | 2 | 0 | 2 | 1 | 0 | 2 | 2 | 0 | 0 | 3 | 2 | 1 | 19 | Moderate |
| 15 | 1 | 1 | 1 | 0 | 0 | 2 | 0 | 2 | 1 | 0 | 0 | 2 | 1 | 1 | 12 | Moderate |
| 16 | 2 | 1 | 1 | 1 | 2 | 2 | 0 | 3 | 1 | 1 | 1 | 1 | 1 | 0 | 17 | Moderate |
| 17 | 2 | 2 | 2 | 2 | 3 | 2 | 0 | 2 | 1 | 1 | 1 | 2 | 2 | 1 | 23 | Severe |
| 18 | 2 | 2 | 3 | 0 | 1 | 2 | 0 | 1 | 1 | 0 | 2 | 3 | 2 | 2 | 21 | Severe |
| 19 | 2 | 2 | 3 | 0 | 2 | 1 | 0 | 2 | 2 | 0 | 0 | 1 | 2 | 1 | 18 | Moderate |
| 20 | 1 | 1 | 0 | 0 | 1 | 0 | 0 | 2 | 3 | 0 | 0 | 1 | 0 | 0 | 9 | Mild |
| 21 | 2 | 2 | 2 | 0 | 2 | 1 | 0 | 2 | 2 | 0 | 0 | 0 | 1 | 1 | 15 | Moderate |
| 22 | 1 | 3 | 3 | 2 | 1 | 2 | 0 | 1 | 1 | 0 | 2 | 1 | 2 | 1 | 20 | Moderate |
| 23 | 2 | 2 | 2 | 0 | 1 | 1 | 0 | 2 | 2 | 0 | 0 | 1 | 1 | 1 | 15 | Moderate |
| 24 | 3 | 3 | 3 | 2 | 2 | 2 | 0 | 3 | 2 | 0 | 2 | 3 | 2 | 1 | 28 | Severe |
| 25 | 2 | 2 | 3 | 1 | 2 | 1 | 0 | 3 | 0 | 0 | 2 | 2 | 2 | 1 | 21 | Severe |
| 26 | 2 | 1 | 3 | 0 | 1 | 2 | 0 | 3 | 1 | 0 | 2 | 2 | 2 | 1 | 20 | Moderate |
| 27 | 0 | 1 | 0 | 0 | 0 | 0 | 0 | 0 | 0 | 0 | 0 | 0 | 0 | 0 | 1 | Mild |
| 28 | 2 | 1 | 1 | 0 | 0 | 0 | 0 | 1 | 1 | 1 | 0 | 0 | 1 | 1 | 9 | Mild |
| 29 | 2 | 1 | 2 | 2 | 1 | 0 | 0 | 2 | 0 | 1 | 1 | 0 | 1 | 1 | 14 | Moderate |
| 30 | 0 | 1 | 1 | 0 | 0 | 0 | 0 | 0 | 0 | 0 | 0 | 0 | 0 | 0 | 2 | Mild |

Table S2. Variables evaluated of intestines condemned by enteritis in the slaughterhouse and frequency distribution by categories.

| Variable | Category | Samples n (%) |
| --- | --- | --- |
| Carcass Condemnation | Yes | 23 (77) |
| No | 7 (23) |
| Visual -only score lesion  (intestines) | Mild | 7 (23) |
| Moderate | 17 (57) |
| Severe | 6 (20) |
| Microscopic classification (lymph nodes) | 1 (Other causes) | 6 (23) |
| 2 (no alteration) | 13 (48) |
| 3 (necrosis/follicular depletion) | 8 (29) |
| Visual -only  classification (intestines) | 1 (colitis) | 19 (63) |
| 2 (serositis) | 5 (17) |
| 3 (coccidiosis) | 2 (7) |
| 4 (other causes) | 4 (13) |
| PCV2 | Presence | 18 (60) |
| Absence | 12 (40) |
| Lawsonia spp. | Presence | 4 (13) |
| Absence | 26 (87) |
| E. coli | Presence | 17 (57)* |
| Absence | 13 (43) |

*Of the 17 E. coli isolates, 16 (100%) presented AMR and 9 (53%) were multidrug-resistant.

Figure S1. Photographs’ examples of the lesion classification of enteritis in the slaughter line. A. Mild classification - segment of small intestine with a focal area of wall hyperemia and serosal edema (arrow); B. Moderate classification - segment of small intestine with fibrinous deposits on the serosal and mesenteric surfaces, associated with edema and hyperemia (arrow); C. Severe classification - segment of the small intestine with diffusely thickened and corrugated serosa (arrow).
